# Robust MS quantification method for phospho-peptides using ^18^O/^16^O labeling

**DOI:** 10.1186/1471-2105-10-141

**Published:** 2009-05-11

**Authors:** Claus A Andersen, Stefano Gotta, Letizia Magnoni, Roberto Raggiaschi, Andreas Kremer, Georg C Terstappen

**Affiliations:** 1 Siena Biotech SpA, Discovery Research, Via Fiorentina 1, 53100 Siena, Italy

## Abstract

**Background:**

Quantitative measurements of specific protein phosphorylation sites, as presented here, can be used to investigate signal transduction pathways, which is an important aspect of cell dynamics. The presented method quantitatively compares peptide abundances from experiments using ^18^O/^16^O labeling starting from elaborated MS spectra. It was originally developed to study signaling cascades activated by amyloid-β treatment of neurons used as a cellular model system with relevance to Alzheimer's disease, but is generally applicable.

**Results:**

The presented method assesses, in complete cell lysates, the degree of phosphorylation of specific peptide residues from MS spectra using ^18^O/^16^O labeling. The abundance of each observed phospho-peptide from two cell states was estimated from three overlapping isotope contours. The influence of peptide-specific labeling efficiency was removed by performing a label swapped experiment and assuming that the labeling efficiency was unchanged upon label swapping. Different degrees of phosphorylation were reported using the fold change measure which was extended with a confidence interval found to reflect the quality of the underlying spectra. Furthermore a new way of method assessment using simulated data is presented. Using simulated data generated in a manner mimicking real data it was possible to show the method's robustness both with increasing noise levels and with decreasing labeling efficiency.

**Conclusion:**

The fold change error assessable on simulated data was on average 0.16 (median 0.10) with an error-to-signal ratio and labeling efficiency distributions similar to the ones found in the experimentally observed spectra. Applied to experimentally observed spectra a very good match was found to the model (<10% error for 85% of spectra) with a high degree of robustness, as assessed by data removal. This new method can thus be used for quantitative signal cascade analysis of total cell extracts in a high throughput mode.

## Background

In order to better understand the vast complexity of the molecular events in biology, good measurement techniques and methodologies are required to investigate the biological processes as they unfold. The presented approach was developed to identify protein targets in Alzheimer's disease as part of the first steps in the drug discovery pipeline. The activated cellular signal transduction pathways were studied in a neuronal disease model immediately upon amyloid-β stimulation[1]. Protein phosphorylation is a well known and extensively used signaling mechanism, so measuring specific changes in protein phosphorylation was used to inspect these pathways. To this end it is required to assess the degree of phosphorylation at a specific protein residue, which differs from the overall degree of phosphorylation of a given protein e.g. observed as a shift in isoelectric point on a gel.

The experimental setup uses stable isotope labeling by normal or heavy oxygen (^16^O or ^18^O) to differentiate between mixed treated and control peptides[2]. This peptide mixture is analyzed by mass spectrometry in a single run. The proteins were extracted and the samples were analyzed in two steps. First the proteins were trypsinized and peptides identified in an MS/MS mode experiment from an unlabeled mixture of the treated and control samples. Secondly the proteins were extracted from the treated and untreated cells, an aliquot split was performed followed by ^18^O/^16^O C-terminal labeling by trypsination in two independent experiments (see Methods). This produced a 'direct' experiment, where the peptides from the treated cells were labeled with heavy oxygen (^18^O) and mixed with peptides from the untreated control cells labeled with light oxygen (^16^O), and an 'inverted' experiment where the labeling was swapped. The samples were subsequently analyzed by mass spectrometry and the acquired spectra were initially processed through a series of analysis steps (see Methods), which are not part of the method presented and therefore not detailed here.

**Figure 1:**
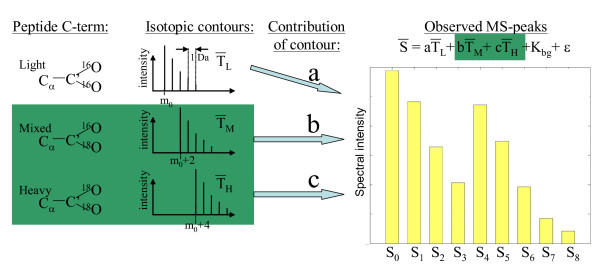
**^18^O/^16^O labeling problem setting**. To quantitatively compare peptides their C-term is ideally labeled with heavy oxygen (two ^18^O) or light oxygen (two ^16^O), but partial labeling also occurs (mixed). These isotope species give rise to overlapping isotopic contours with individual contributions (a, b and c) to the observed MS spectra peaks. The input data used for the data analysis were nine observed spectral intensities (S_0_-S_8_) including possible missing values. These intensities () are assumed to equal a weighted sum of three isotope contours (Peptide C-terms:  Light (^16^O_2_),  Mixed (^18^O, ^16^O) and  Heavy (^18^O_2_)), a constant background K_bg _and an error term ε. The weights (a, b and c) are of particular interest since they describe the contribution of unlabelled (Light:a) and labeled (Mixed:b and Heavy:c) isotopes. In the theoretical case where the background constant and error term are removed a theoretical framework is presented (see Table 1), where the above experiment is performed twice for each peptide with swapped labels (i.e. Direct and Inverted experiments).

The problem setting addressed here starts from a set of label swapped pairs, each with up to 9 spectral intensities (see Figure 1) extracted from a large range of MS spectra summing ion counts from multiple charge states and an extended retention time. The choice of using up to 9 peaks (missing values were allowed) in the quantitative MS analysis was a pragmatic one, since in most spectra the 9^th ^peak is already within the noise range. A set of inherent problems to the ^18^O labeling technique are treated here: one is the overlap of three isotopic contours from the labeled and unlabeled peptides; another is the non-perfect labeling efficiency, which along with experimental noise needs to be taken into account in order to get as robust and reliable quantitation as possible (see Figure 1).

**Table 1:** Overview of Method Variables

**Direct labeling:**	Light (^16^O_2_)	Mixed (^16^O, ^18^O)	Heavy (^18^O_2_)
Treated (Lab. pep.):			
Control (Unlab. pep.):			
Intensities: *I*_*D*_	*a*_*D*_	*b*_*D*_	*c*_*D*_
**Inverted labeling:**	Light (^16^O_2_)	Mixed (^16^O, ^18^O)	Heavy (^18^O_2_)
Control (Lab. pep.):			
Treated (Unlab. pep.):			
Intensities: *I*_*I*_	*a*_*I*_	*b*_*I*_	*c*_*I*_

The theoretical quantities used to derive the ratio between treated and control samples are listed. The desired intensities for treated and control samples were calculated from the estimated intensities (see Eq. 2II–III) resulting from the multivariate linear regression fit after normalization (*a, b *and *c*). The total intensity of a spectra *I = a + b + c*, where in the table subscripts *D *and *I *are added referring to the Direct and Inverted experiments, respectively, and the superscripts *T *and *C *are added referring to treated and control samples, respectively.

We have chosen to report the primary end result as the fold change between treated and control. Choosing the fold change also means that the absolute intensities of treated and control are no longer needed, thus allowing the assumptions used to be reduced to one i.e. equal labeling efficiency between the two label swapped samples (see Methods).

The theoretical probability distribution between double, single and no incorporation of ^18^O upon labeling can also be derived[3]. Here we have chosen not to make any such assumptions, but rather use additional experimental data in the form of label swapping and the limited assumption of equal labeling efficiency of a given peptide upon label swapping.

The basic analysis methodology starts with the fitting of theoretical isotopic contours for the heavy, mixed and light isotopes to the observed signal (see Figure 1). The fitting was carried out using multivariate linear regression where the squared error was minimized[4] to yield the three intensities of labeled and unlabeled isotopes with their respective confidence intervals (see Methods). To reflect the quality of fit of the multivariate regression onto the resulting fold change, confidence intervals were calculated by a parametric bootstrap using the estimated covariance matrix of the three regression coefficients.

Some previous methods have relied on just a few peaks (2–4 peaks) to estimate the ratio between treated and control[5,6] and in most cases the peptide sequence was assumed unknown. More relevant information is present in the remaining peaks which can be utilized to improve the peptide abundance estimate. The abundance estimate can be improved further if the peptide sequence and post-translational modifications are known, since the theoretical isotopic contour is improved, as presented here. The presented method can also use estimated isotopic contours directly if the peptides have not been identified prior to MS quantitation[7,8]. This is the case for a method recently published by Eckel-Passow et al. which uses averagine (an imaginary average amino acid) and all of the MS-peaks presently discernible from background noise[3,9]. They used the linear regression described by Mirgorodskaya et al.[10] which is analogous to the one presented here to calculate the isotopic intensities. The sample used for their analysis was a simple mixture of two proteins in a 1:1 ratio, in contrast to proteins originating from a complete cell lysate as investigated here and by others[2,11-13].

To assess a new peptide quantitation methodology ideally you would need to have a large and varied set of spectra with already known abundances. In short of such a data set we propose to use simulated spectra for a thorough model assessment and the limited experimental data for validation.

## Results

**Figure 2:**
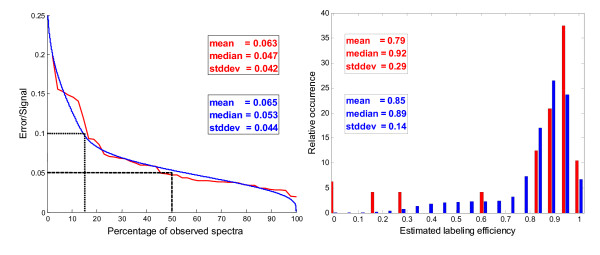
**Simulated data mimicking experimental distributions**. **a) **The error to signal ratio (ε/S-ratio) displayed shows that 85% of the experimentally observed spectra (red line) matched the theoretical reconstruction well, with less than 10% of the total spectral intensity assigned to the error term (dotted line). 50% of the spectra were matched very well with less than 5% error (dashed line). Noise was introduced into the simulated spectra in order to reflect a similar ε/S-ratio distribution (blue line). **b) **The labelling efficiency was found to vary considerably between experimental spectra (red bars). One peptide was not labelled at all, while another was completely absent in the treated sample, which produced the three spectra with a labelling efficiency of zero observed. The simulated spectra were generated to reflect this long tailed and high variability distribution (blue bars).

To perform an in depth assessment of the methodology we first present a characterization of some important parameters on the experimentally observed spectra. This characterization was used to generate similar spectra *in-silico *where desired parameters could be imposed and compared to the ones estimated by the model after adding noise. The simulated spectra were generated in order to reflect, as much as possible, the experimentally observed spectra by using the same peptides and by mirroring the distributions observed for a set of important observable parameters. One important parameter is the proportion of total spectral intensity which cannot be explained by the model's three overlapping isotopic contours and remains as residuals or errors, here termed the ε/S-ratio (). The error term contains experimental noise as well as any mismatch between theory (including data fitting) and practice. The data agreed very well with the model as shown in Figure 2a with less than 10% error for 85% of the spectra and less than 5% error for 50% of the spectra.

Noise was added to the simulated data with a distribution similar to the one found for the experimental spectra, thereby reflecting the experimental data from this perspective, resulting in less than 10% error for 85% of the spectra and less than 5% for 45% of the spectra. Another important parameter is the labeling efficiency which is defined as the ratio between the labeled isotope intensity (either fully or partially labeled) and the total intensity of the sample undergoing labeling (see Figure 2b and Equation 2). In most of the spectra a reasonably good labeling efficiency is observed (above 0.6), while there are three spectra pairs with low or absent labeling. We found that it was unrealistic to rescue these spectra with label efficiencies close to zero (data not shown) so for the simulated spectra the tail of the imposed label efficiency distribution was cut off at 0.4. The labeling efficiency shown for the simulated spectra is the one estimated by the model in the same manner as it is done for the experimentally observed spectra, which naturally differs from the one imposed when constructing the spectra. Finally in supplementary Figure [see Additional file 1] the estimated fold change distribution is shown for the experimental and simulated spectra. The average fold change was close to one, as one would expect from a biological perspective since only a small part of the phosphorylated proteins were expected to be involved in the cellular response. The experimental spectra had a trend towards positive fold changes, which we chose not to impose on the simulated data. Furthermore we also chose a larger spread of the fold change to provide enough data points for a statistical analysis of larger fold changes, which actually increase the average fold change error as shown below. Having inspected the experimentally observed spectra and ensured that the simulated spectra reflect their overall measurable characteristics, we can perform an in-depth assessment of the presented model's behavior using simulated spectra followed by an investigation of the experimentally observed spectra.

### Fold change assessment

The fold change parameter reports the change in abundance between the treated and control samples in a symmetrical and straightforward manner by a simple ratio (see Methods). We used the simulated data presented above to assess the model estimates of fold change and its dependencies on noise, label efficiency and the absolute value of the fold change itself. We have chosen to display primarily the relationships with respect to the estimated parameters as they can be calculated from the experimentally observed spectra and thus relate the fold change to them.

As the noise level goes up the difference between true fold change and estimated fold change increases, but since the noise is distributed over 9 observable peaks, which are fitted in unison by the model, it is not directly clear how the estimated fold change would behave. To measure the quality of the estimated fold change we introduce the fold change error defined below as the difference between true (or imposed) fold change and the fold change estimated from the model based on the spectra:

(9)

**Figure 3:**
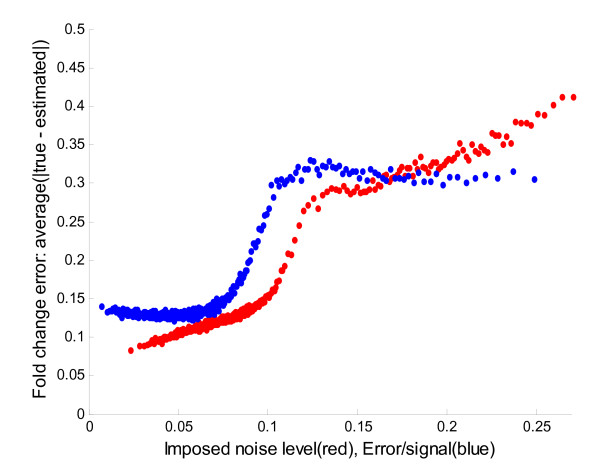
**Fold change quality assessment**. To assess the quality of the estimated fold change the average error was calculated as a function of the imposed noise level (red) and the observable error to signal ratio (ε/S-ratio in blue) calculated as part of the model fitting. The sigmoid shape of the ε/S-ratio reflects that when the imposed noise fits the model (low ε/S-ratio) the fold change error does not decrease, and when the imposed noise doesn't fit (high ε/S-ratio) the fold change error remains level. The observation that the ε/S-ratio (blue) is flat at the ends of the sigmoid reflect the fact that by chance, for low ε/S-ratios the noise accidentally fits with the isotopic contour, thus increasing the fold change error. The flat end for high ε/S-ratios is just the inverse i.e. by chance the noise pulls in opposite directions and cancels out in the linear regression fit, thus lowering the fold change error while having a large ε/S-ratio.

In Figure 3 the fold change error is plotted as a function of the imposed noise level and the ε/S-ratio originating from the model residuals after fitting the three overlapping contours to the spectra. The imposed noise level and ε/S-ratio are correlated with a correlation coefficient of 0.80, which shows that the ε/S-ratio is a reasonably good indicator of the level of noise in a spectrum.

**Figure 4:**
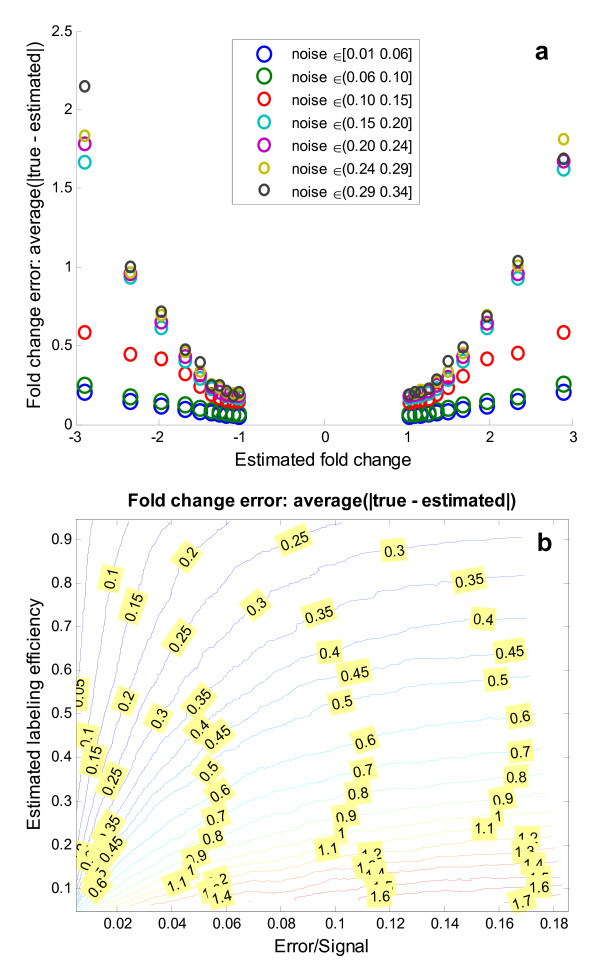
**Fold change error estimation from observable parameters**. **a) **Large estimated fold changes were found to entail increasing absolute error in the fold change estimate. This can be explained from the observation that large fold changes are the result of one sample (either treated or control) being present in low amounts which in turn increases the influence of noise and poor labeling efficiency. For low degrees of added noise (noise ∈ [0.01 0.10]) the relationship is strikingly linear, for higher noise levels the fold change error is seen to increase more rapidly. **b) **The contour plot shows which fold change error can be expected (contour lines) for a given Error/Signal-ratio and estimated labeling efficiency (x- and y-axes). Since the two latter parameters are estimated by the method for experimentally observed spectra as well, the plot can be used to look up what would be the expected fold change error given an estimated labeling efficiency and an estimated error/signal ratio.

**Figure 5:**
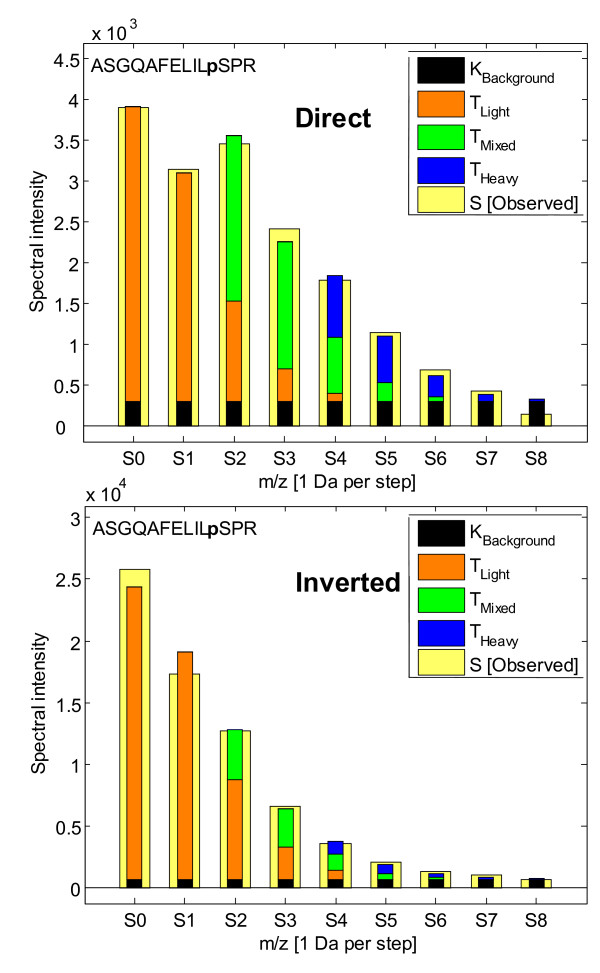
**Experimental label swapped spectra pair**. Fig. 5 The two label swapped spectra pairs are shown (Direct and Inverted) for the peptide ASGQAFELIL**p**SPR. The observed spectra S (yellow bars) is shown along with the model reconstruction using the three contributing isotopic contours (T_Light_(orange), T_Mixed_(green) and T_Heavy_(blue)). The labeling efficiency for this particular peptide was rather low: 0.61, but due to the clear signal it was possible to estimate a fold change of 2.49 [2.05 3.21]. The ε/S-ratio was 0.06 reflecting a model fit around average for the experimental spectra. The spectral intensity was found to vary considerably between the direct and inverted label swapped experiments (average peak intensities: <S_D_> = 1899 and <S_I_> = 7880), which is likely to have originated primarily from variation in phospho-peptide enrichment.

The fold change estimate was found to depend on the labeling efficiency [see Figure Additional file 1], but even at low labeling efficiencies (<60%) the method yielded reasonably good fold change estimates at moderate noise levels (noise < 0.1). The imposed labeling efficiency and the one estimated after added noise were highly correlated with a correlation coefficient of 0.94, showing that the model estimate is quite reliable [see scatter plot in Additional file 1]. Similarly the fold change error increased with increasing fold change as shown in Figure 4a, but in a very noise dependent manner where a small linear response was found for low degrees of noise (noise < 0.1), while higher noise ranges result in drastic increases in average fold change error. This is an argument in favor of suggesting a spectral quality threshold around ε/S-ratio < 0.1. The estimated fold change had a high correlation coefficient of 0.91 with the imposed fold change validating the presented methodology over a wide and representative range of labeling efficiencies and noise levels. The interconnected influence of labeling efficiency and noise on the fold change error is not straightforward (see Figure 4b), but is relevant in the experimental setting. For example it should be noted that at low noise ranges (noise < 0.05) a reasonably low fold change error, can be obtained even down to mediocre labeling efficiencies (Lab.Eff. > 0.6), as illustrated in Figure 5. To provide adequate sampling the contour plot is made using a separate data set with flat distributions for all parameters.

### Fold Change Confidence Interval Assessment

**Figure 6:**
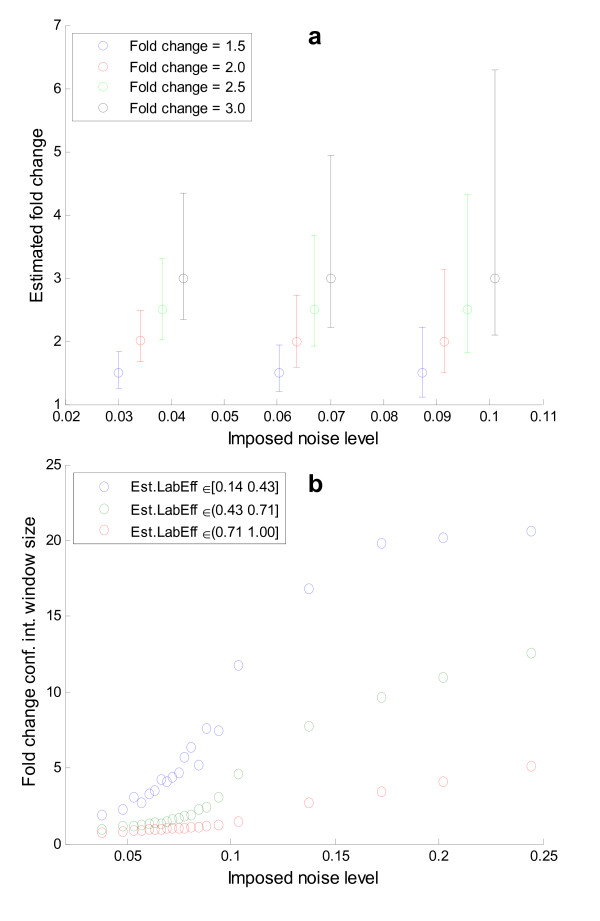
**Assessment of the fold change confidence interval**. **a) **To assess the fold change 95% confidence interval estimate it has been plotted for a subset of fold changes and noise levels as shown. The confidence interval was seen to tighten up when the noise level decreases, which illustrates its value as an assessment parameter of spectral quality. The asymmetry in the confidence interval reflects how the uncertainty and multivariate dependencies between the estimated isotope intensities influence the fold change estimate. Furthermore the confidence interval widens when the fold change increases, in accordance with the increased fold change error (see Fig. 4). **b) **The confidence interval was also found to mirror the uncertainty caused by noise and low labeling efficiency, thus in total capturing important aspects of spectral quality.

In a high throughput setting it is not possible to assess the quality of each individual spectra pair and of the model fit manually, so if only a fold change is reported the spectral quality aspect is missing. To this end we have computed the confidence interval for each reported fold change using a bootstrap based on the regression (see Methods). The relevance of the confidence interval lies in its ability to reflect the quality of the spectra pair. This means that for good spectra the confidence interval should be narrow, while for poor spectra it should widen up. A subset of fold change values and noise levels were extracted to illustrate how the fold change confidence interval tightens around the estimated fold change when the noise level decreases (see Figure 6a). The quality of the spectra depends primarily on the level of imposed noise and imposed label efficiency, which was found to correlate well with the fold change confidence interval window size (see Figure 6b). The estimated 95% confidence interval around the fold change actually contained the imposed fold change 96% of the cases showing that estimate is highly reliable.

### Consistency Check

Based on the obvious assumption that a contributing intensity cannot be negative we were able to derive a set of constraints in order to assess whether a label swapped spectra pair was mutually consistent (see Methods). They specify that *a*_D _≥ *b*_*I *_+ *c*_*I *_and *a*_*I *_≥ *b*_*D *_+ *c*_*D*_, which basically means that a sample may not change intensity upon label swapping. They are important in order to identify problematic spectra pairs and were used as an initial filter on the experimental and simulated spectra pairs, thus flagging 8% and 5%, respectively.

### Examples from Experimental Spectra

To illustrate the reported results on experimentally observed spectra pairs, a peptide with low labeling efficiency (0.61) is shown in Figure 5. Nevertheless a tight fold change interval could be estimated where FC = 2.49 with a 95% confidence interval of [2.05 3.21]. Another medium intensity peptide was observed as two separate species, sharing a phosphorylation site (pTPGTPGpTPSYPR and TPGTPGpTPSYPR). Using the presented methodology the phosphorylation site specific fold change was estimated for the shared Thr phosphorylation to 1.33 with a 95% confidence interval of [1.17 1.64] (see Methods). This peptide originates from the microtubule-associated protein 2 (map-2), the phosphorylation state of which has been found to influence cytoskeleton structure[14]. In Alzheimer's, the disease studied, cytoskeleton integrity is known to be of great importance[15].

### Robustness Assessment

**Figure 7:**
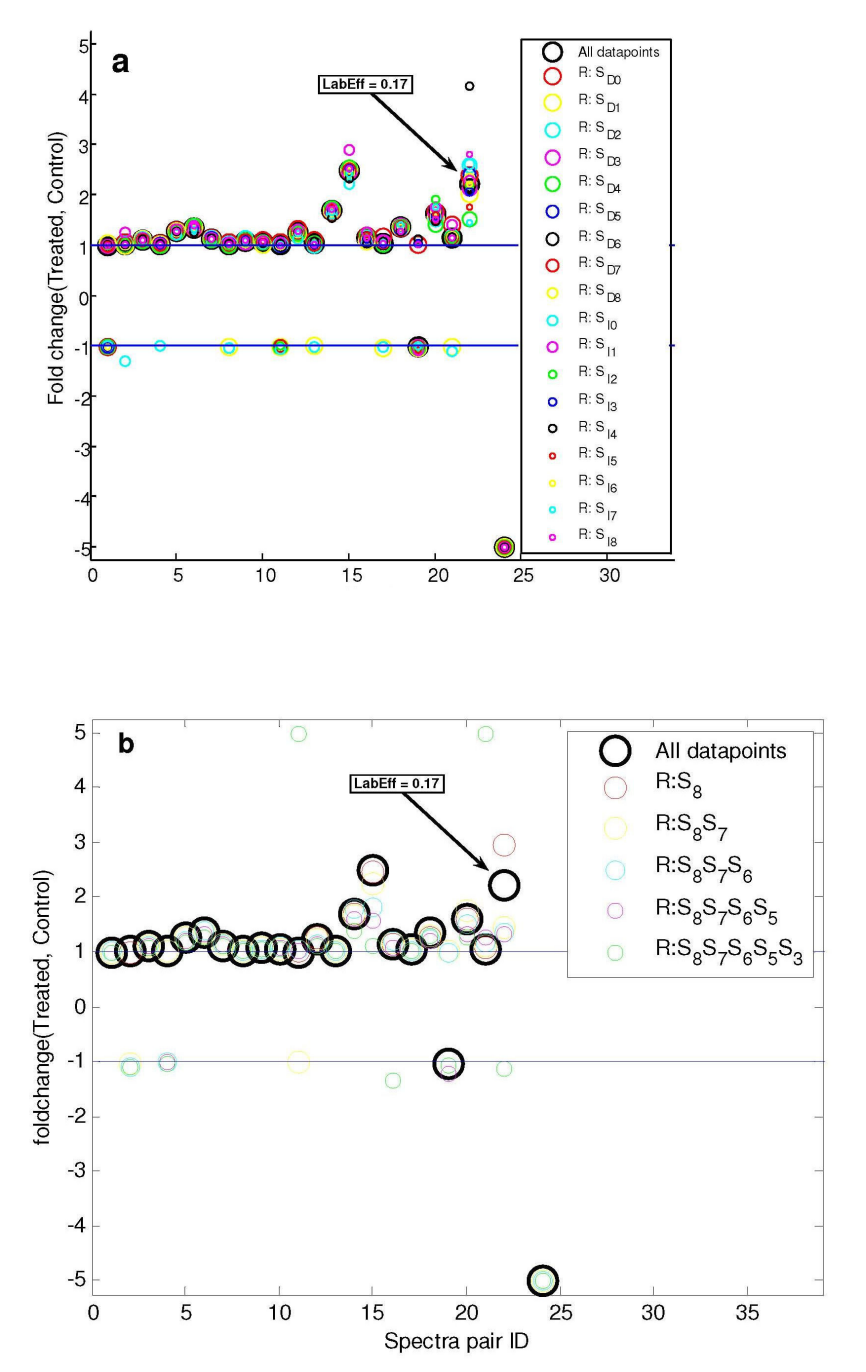
**Robustness assessment of the fold change estimate**. **a) **To assess the robustness of the model's fit to the observed spectra, one data point (Direct:S_D0_-S_D8_, Inverted:S_I0_-S_I8_) at a time has been removed and the resulting fold change recalculated. The graph shows for each experimentally observed spectra pair (x-axis) how the estimated fold change varies when one of the spectra peaks is missing. Overall only small degrees of variation upon data removal were observed, thereby demonstrating the redundancy of the spectra and robustness of the fold change estimate. The highlighted case where the fold change varies considerably is due to very low labeling efficiency (LabEff). A fold change saturation limit of +/- 5 was imposed for proper visualization. **b) **To challenge the method's robustness even further multiple data points have been removed. This has been done by removing peaks in pairs starting from the on-average least intense (R:S_8 _means removing S_D8 _and S_I8_) and moving up (R:S_8_S_7 _means removing S_D7_, S_I7_, S_D8 _and S_I8_) etc. Overall it is seen that peptides with a high fold change tend to be more susceptible to missing values. The method appears to handle up to four missing values well, but in most of the observed cases even six missing values result in reasonable fold change values.

To assess the quality of the presented method directly on the experimentally observed spectra we performed a robustness test. In Figure 7 one spectra peak was removed at the time and the spectra pair re-analyzed to show the resulting fold change. This is also based on the recognition that occasionally some peaks cannot be measured or are contaminated with interfering species and was feasible by utilizing the inherent redundancy contained within the spectra. The fold change estimates were found to be very robust towards the removal of single peaks and outliers could in this manner be identified to support the removal of interfering isotope species, thereby improving the final fold change estimate.

## Discussion

The presented method can estimate residue specific protein phosphorylation fold changes with associated confidence intervals from an ^18^O/^16^O label swapped spectra pair independently of the label efficiency, if minimal labeling occurs. The only prerequisite for the presented methodology is equal peptide specific label efficiency between label swapped experiments. The fold change for each peptide had a very low average and median error of 0.16 and 0.10, respectively, where the error was estimated from simulated spectra with similar noise and labeling efficiency distributions as the experimentally observed spectra. The labeling efficiency and spectrum noise were along with the absolute value of the fold change itself found to be the main determinants of fold change error, but are being continuously improved[16]. Using the simulated data we were able to map this relationship in a contour plot, where the average fold change error is reported, given the estimated labeling efficiency and ε/S-ratio. The contour plot displays the non-linear relationship between labeling efficiency and ε/S-ratio and allows an additional estimate of the fold change error directly applicable to experimental observed spectra. A qualitative decrease in fold change estimation was found for a few poor spectra with an ε/S-ratio above 0.1 or estimated labeling efficiency below 0.5, which had an average fold change error of 0.50. If these spectra are excluded the average and median fold change drops to 0.13 and 0.09, respectively. Two major drawbacks where highlighted by Miyagi and Rao[17] of the ^18^O/^16^O-labeling technique: variability in labeling and computational tools, both are addressed by the presented method.

## Conclusion

The method presented was developed to support its application in high throughput experiments by quality check filtering based on spectra pair consistency and the accurate reporting of fold change confidence interval. The fold change confidence interval was found to summarize spectral quality nicely from several aspects such as noise, labeling efficiency and sample signal. We found that the confidence interval provided very valuable information, thus reducing the amount of time spent quality checking the underlying spectra manually. A matter of experimental reality is the occasional intrusion of an interfering species into a spectrum, which can be identified and eliminated using the leave-one-out robustness assessment presented. The described method can also be used for quantitation in a broader context such that different peptide isotope contributions (due to post-translational modifications or peptidase miscleavages) can be joined to yield a more accurate overall peptide fold change estimate and resulting protein quantitation. The presented method is thus useful for the elucidation of the constitution and dynamics of cellular signaling pathways by allowing the accurate measurement of residue-specific phosphorylation events.

## Methods

### Experimental Protocol

Rat cortical neurons were treated with amyloid-β for a duration of 5 minutes and compared to untreated controls in order to identify proteins with differentially phosphorylated residues[18]. The peptide labeling technique uses heavy (H_2_^18^O) and normal (H_2_^16^O) water as described by Yao et al.[19] in a similar manner to others [2,11]. This should ideally label all peptides except those originating from the C-terminal peptide of the protein. An aliquot split was performed in the biological sample and label swapping was performed in order to separate label efficiency from biological variation. The phosphorylated peptides were enriched using the Phos-Select IMAC resin (Sigma-Aldrich), separated using 2D-nanoflow-liquid chromatography with SCX step gradient as first dimension and RP as the second and finally analyzed using a nanoflow-ESI-QTOF-MS. The intensity of the isotopes of each peptide species considered was measured from MS spectra acquired in profile mode. All peptide spectra with an ion count above 40 were combined and centroided using the Mass Measure tool in MassLynx 4.0 (supplied by Waters), summing a window of retention time and multiple charge states. The isotope Modeling tool of MassLynx was used to compute the theoretical contour based on the identified peptide sequence and its post-translational modifications. The experiments produced a total of 52 spectra of which 4 were found to be inconsistent (see Label Swapped Spectra Pair Consistency) resulting in a total of 48 spectra pairs used in the presented analysis. All data are available as a table in the supporting material [see Additional file 2].

### Simulated Data

The present section describes the details needed to reproduce the data presented in the Result section covering noise level, labeling efficiency and fold change calculations. One million spectra were generated of which 53860 were found to be inconsistent (see Label Swapped Spectra Pair Consistency) and removed, thereby leaving 946140 which were characterized in Figure 2, 3 and 4. A simulated spectra for a peptide was generated by adding noise (here denominated imposed noise) to the sum of three idealized isotope contours (corresponding to light, mixed and heavy isotopes). The imposed noise was added from two flat distributions, since this was found to give an ε/S-ratio distribution similar to the one observed for the experimental spectra (see Figure 2). One distribution had a maximum of 37% noise and the other a maximum of 14% noise. The simulated spectra were generated by first choosing one of the two distributions for a spectrum e.g. 14%, then generating a random number for each peak ranging from +14% to -14% and adding this number to each peak in the spectra. This imposed noise level reported for each spectrum was the actual noise added by the random number generator, which varies from one simulated spectrum to another. The idealized spectra used were the same as those generated as part of the analysis of the 48 experimental spectra pairs used as example data.

### Multivariate Linear Regression

The multivariate linear model fits three (light, mixed and heavy) stepwise shifted identical theoretical isotopic contours (T_0_–T_4_) to an observed spectrum (S_0_–S_8_) with intensities a, b and c finding the least squares solution[6] (see Figure 1). The model also includes an adaptable background level (K_bg_) resulting in a total of four parameters estimated from nine observables. An adaptation of the multivariate regression algorithm was required in order to produce realistic (non-negative) isotope intensities. If an estimated isotope intensity parameter (a, b or c) came out negative the regression was redone while fixing it to zero. In Table 1 an overview is given of the variables used. The statistical analyses were performed in MatLab and scripts are available upon request.

### Label Efficiency Subtraction

The labeling reaction yield has been observed to vary considerably between the various phospho-peptides studied. Some have a very high labeling reaction yield, most are labeled well and yet some don't seem to be labeled at all [see Additional file 3]. With the single assumption of equal labeling reaction yield upon label swapping we derive the ratio between treated and control, which is all that is needed to calculate the fold change and is independent of the labeling efficiency of the peptide in question. Naturally when the labeling efficiency becomes very low or absent the fold change can no longer be estimated properly, but this should be reflected in the fold change confidence interval.

Using the primary assumption of equal labeling efficiency (explicitly defined in Eq. 2I) we can derive the ratio between treated and control using theoretical quantities, which reads:

(1)

Where Table 1 defines the theoretical quantities used and Eq. 2II–III describes how the estimated coefficients from the multivariate linear regression relate to the theoretical quantities. The primary assumption used is that the label reaction yield remains unchanged between direct and inverted experiments (see Equation 2I). In Eq. 2II–III we describe the expected value of the estimated coefficients in relation to the theoretical quantities. Furthermore the estimated coefficients are normalized to ensure equal peptide intensities upon label swapping (required by the theoretical framework).

(2)

Eq. 2 **(I) The primary assumption of this paper is defined here and states that the peptide label efficiency remains unchanged between Direct and Inverted experiments (i.e. label swapping). (II and III) The coefficients (*a*, *b *and *c*) used in the theoretical framework (see Table **1**) are derived from the estimated coefficients by normalization, such that the total intensities (*I*_*D *_and *I*_*I*_) between Direct and Inverted experiments are equal. The expectancy, *E()*, of an estimated parameter is its theoretical counterpart after normalization. See Table **1** for an explanation of the theoretical quantities used**.

The ratio is all that is needed in order to calculate the fold change (see Equation 3) and is independent of the peptide specific labeling efficiency.

The number of assumptions incorporated into the model has been minimized to avoid inconsistencies due to noise from the experimental measuring process. An example is the labeling efficiency which could have been formulated in a much more constraining manner, where the proportion of each isotope species (heavy, mixed and light) would have been assumed unvaried between the two label swapped experiments. In effect when noise is present in the experimental data or in the simulated data they rarely conform to this demanding assumption (data not shown).

### Definition of Fold Change

The fold change definition used (see Equation 3) describes in a symmetric way the change in abundance between treated and control.

(3)

Eq. 3 **The fold change definition applied ensures a symmetrical representation of an increase in abundance from control to treated (e.g. 1.25 means 25% increase) with respect to a decrease in abundance (e.g. -1.25 means 25% decrease). The ratio between treated (I^T^) and control (I^C^) intensities was estimated using Equation (1)**.

### Fold Change Confidence Interval Estimation

The basic assumption used for the confidence interval calculation of the fold change was that the parameters estimated by the linear regression (a, b and c) were following a multivariate Gaussian distribution. Although this is known not to be exact it is a workable solution which upon sufficient sampling becomes reliable. The regression fit method used to fit MS spectra was as described by Chatterjee and Hadi[6] which also estimates the covariance matrix ∑ of the returned parameters (a, b and c). Subsequently a bootstrap calculation was performed in order to estimate the fold change distribution. 10000 random values (a_k_, b_k _and c_k_) were drawn from a multivariate Gaussian distribution with mean vector [a, b, c] and covariance matrix ∑; for each random set (a_k_, b_k _and c_k_) the fold change FC_k _was calculated using Equations 1 and 3. The 95% confidence interval was estimated by making use of the bootstrap generated fold change distribution[20].

### Label Swapped Spectra Pair Consistency

It is obvious that all intensities must be positive, which was imposed as part of the regression for a, b and c, but when a label swapped spectra pair was analyzed the intensity of the "labeled" sample which remains unlabeled ( and ) remains unconstrained and was in some cases found to be negative. By imposing the obvious constraints:  and  we can analytically derive the resulting constraints for the observed regression parameters:

(4)

These are also quite obvious constraints: Equation 4I requires that the intensity of the unlabeled isotope in the direct experiment *a*_*D *_cannot be less than the intensity of the labeled isotopes in the inverted experiment *b*_*I*_*+c*_*I*_. This follows from the fact that *a*_*D *_contains the entire control sample as well as what didn't get labeled of the treated sample, while *b*_*I*_*+c*_*I *_only upon perfect labeling maximally can contain the entire control sample. Equation 4II is the symmetrical version for the treated sample. If these constraints are not fulfilled after normalization then the estimated parameters from the two spectra are not mutually consistent. This has been implemented as a check which was applied throughout in order to flag and filter out spectra pairs. This was the case for 13% of the experimentally observed spectra and 5% of the simulated spectra. As part of further developments we propose to develop a method to rescue these spectra e.g. by realigning the parameters (*a*, *b *and *c*) in order to fulfill the constraint. In any case the flagging of inconsistent spectra would remain an important quality indicator. Another way of looking at these constraints puts quite interestingly upper and lower bounds on the treated versus control ratio:

(5)

### Phosphorylation Site Specific Fold Change Estimation

The phosphorylation site specific fold change can be derived from the treated versus control ratios (TC-ratio) of all the peptide species containing the phosphorylation site in question. These different peptide species may be observed due to miscleavages by the peptidase, two phosphorylations on the same peptide, or other post-translational modifications, which split the observed peptide in two ore more distinct isotope species. The objective is, as above, to estimate the overall TC-ratio, in this case involving intensities from all the observed peptide isotope species containing the phosphorylation site in question, this can be formulated as:

(6)

where *I*_*i*_^*T *^is the theoretical intensity of the MS unique peptide (i.e. m/z and retention time unique) number *i *and is a shorthand where the Direct/Inverted label symbol is left out e.g. *I*_*i*_^*T *^= *I*_*D*, *i*_^*T *^= *I*_*I*, *i*_^*T*^, since the two are identical. The estimate of the treated and control intensities required here can be directly taken from our normalized intensities described above, since the normalization step applied retains the scale (see Equation 2 II and III). Actually the normalization used aligns the theoretical intensities to the average of their expected estimated coefficients such that: . This means we can use the TC-ratio to derive the desired intensities of the treated and control samples for a given peptide isotope:

(7)

(8)

When multiple peptides are joined to yield a single fold change an added experimental assumption is made of equal detectability of the involved peptide species. While for ^18^O labeling the change is believed to be negligible it is known not always to be so between different peptide species in general as described by Tang et al.[21].

### Visualization statistics

Generally in the figures reported we have used bin counting for optimal visualization. This means that when x versus y is plotted, each spot or circle reports the average x- and average y-value for a given x-axis percentile of the data e.g. if there are 50 spots the first spot reports the average x, y-value for the 2% of data with the lowest x-values. This enables a good estimation of the x-, y-values and also shows the data distribution without flooding the plot with each individual x, y pair. In the special case of reporting fold changes the mean and standard deviation were calculated on "zero-centered fold changes" (*zero centered fold change *∈ (-∞, +∞)) in order to perform the statistics on a continuous value set i.e. before calculation of mean or standard deviation, one was added to all fold change values below -1 and one was subtracted from all the others. After calculation of the mean it was transformed back into a real fold change (*fold change *∈ (-∞, -1) ∪ (1, +∞)) for reporting or visualization.

## Authors' contributions

CA conceived the original idea, performed the bioinformatics analyses and wrote the manuscript. SG performed the MS experimental work, provided the processed data to be used in the calculations, wrote the experimental details related to the MS analysis and MS data processing, and contributed to the manuscript revision. LM gave statistical support and contributed to the manuscript revision. RR participated in the design of the study, evaluation of results and helped to draft the manuscript. AK contributed to discussions throughout the study, critically revised the current manuscript and was involved in its final approval. GCT contributed to discussions that led to the initial design of the study, critically revised the current manuscript and was involved in its final approval.

## Supplementary Material

Additional file 1 **Table containing experimental and related data**. The file 'MethodQuant_Additional_data_pep_S_T.xls' is lists the peptide sequence with phosphorylation sites indicated by a lower case p, the experimentally observed spectra peaks S0–S8 and the theoretical isotopic contour of the peptide T0–T4. Click here for file

Additional file 2 **Striking figure: Fold change error contour with examples**. The file 'MethodQuant_Striking_figure.ppt' is shows experimental examples from various areas of the labeling efficiency and error/signal landscape depicted in Figure 4b. Click here for file

Additional file 3 **The file 'Method MSquant_supporting material.doc' is containing supplementary figures referenced in the text**. Click here for file
