# Supplementary material for: Robust MS quantification method for phospho-peptides using 18O/16O labeling
Source: BMC Bioinformatics. 2009 May 11;10:141. doi: 10.1186/1471-2105-10-141 (PMC2693437; doi:10.1186/1471-2105-10-141)
Supplement: Additional file 2 — Striking figure: Fold change error contour with examples. The file 'MethodQuant_Striking_figure.ppt' is shows experimental examples from various areas of the labeling efficiency and error/signal landscape depicted in Figure 4b. [file 1471-2105-10-141-S2.ppt]

## Slide 1
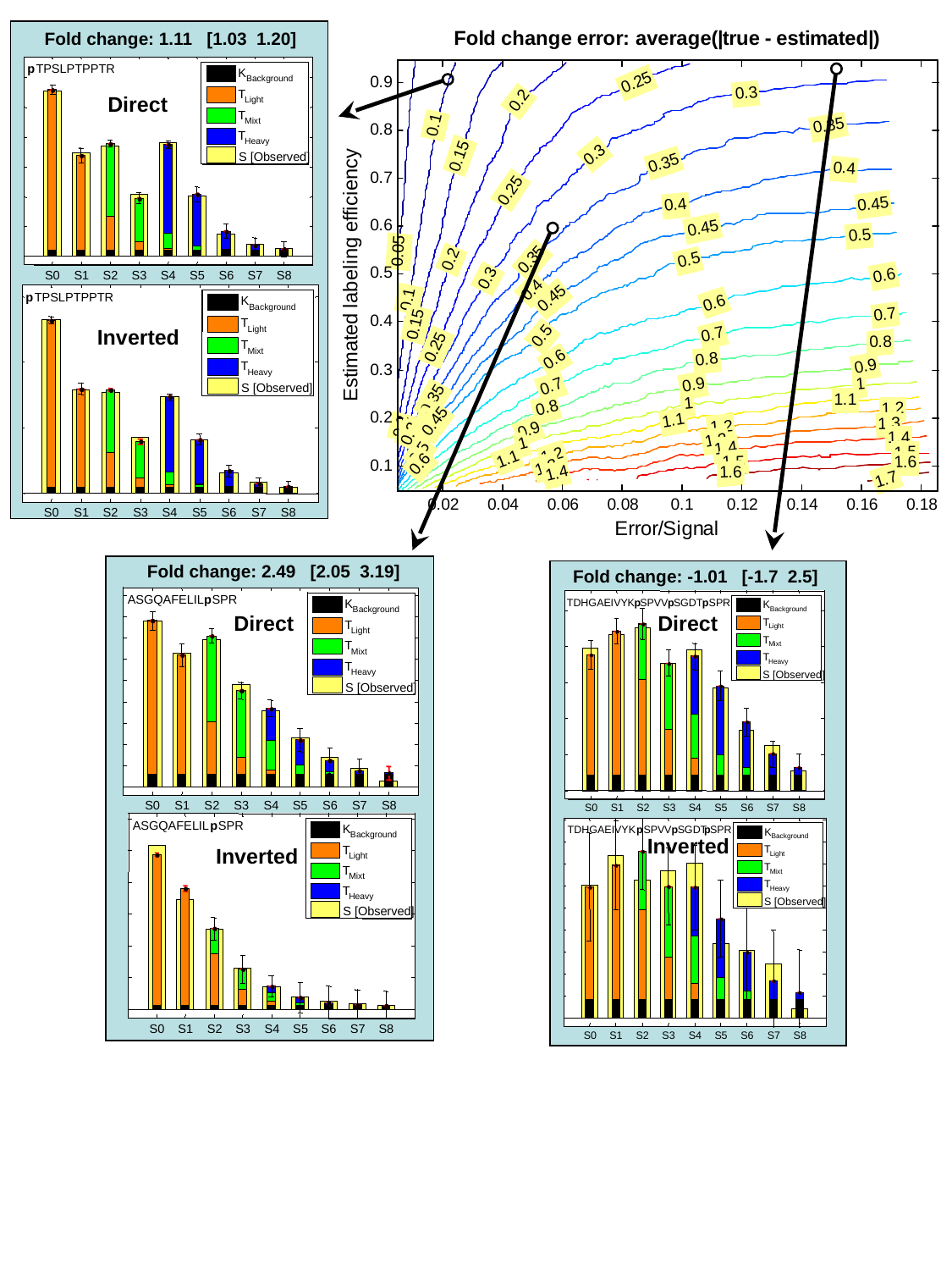

Fold change: 1.11 [1.03 1.20]
p
TPSLPTPPTR
K
Background
T
Direct
Light
T
Mixt
T
Heavy
S [Observed]
S0
S1
S2
S3
S4
S5
S6
S7
S8
m/z [1 Da per step]
p
TPSLPTPPTR
K
Background
T
Inverted
Light
T
Mixt
T
Heavy
S [Observed]
S0
S1
S2
S3
S4
S5
S6
S7
S8
Direct
Inverted
Fold change: 2.49 [2.05 3.19]
ASGQAFELIL
p
SPR
K
Background
T
Light
T
Mixt
T
Heavy
S [Observed]
S0
S1
S2
S3
S4
S5
S6
S7
S8
Direct
ASGQAFELIL
p
SPR
K
Background
T
Light
T
Mixt
T
Heavy
S [Observed]
S0
S1
S2
S3
S4
S5
S6
S7
S8
Inverted
Fold change: -1.01 [-1.7 2.5]
TDHGAEIVYK
p
SPVV
p
SGDT
p
SPR
K
Direct
Background
T
Light
T
Mixt
T
Heavy
S [Observed]
S0
S1
S2
S3
S4
S5
S6
S7
S8
TDHGAEIVYK
p
SPVV
p
SGDT
p
SPR
K
Inverted
Background
T
Light
T
Mixt
T
Heavy
S [Observed]
S0
S1
S2
S3
S4
S5
S6
S7
S8
