# Supplementary material for: Robust MS quantification method for phospho-peptides using 18O/16O labeling
Source: BMC Bioinformatics. 2009 May 11;10:141. doi: 10.1186/1471-2105-10-141 (PMC2693437; doi:10.1186/1471-2105-10-141)
Supplement: Additional file 3 — The file 'Method MSquant_supporting material.doc' is containing supplementary figures referenced in the text. [file 1471-2105-10-141-S3.doc]

**Robust MS Quantification Method for Phospho-peptides using ^18^O/^16^O Labeling**

**Claus A. Andersen, Stefano Gotta, Letizia Magnoni, Roberto Raggiaschi, Andreas Kremer and Georg C. Terstappen**

Siena Biotech SpA, Discovery Research, Via Fiorentina 1, 53100 Siena, Italy

# Supporting material

Figure S1 The degree to which a peptide is labeled (the labeling efficiency) varies dramatically between peptides. On the left is a special case where a peptide has been fully labeled (full labeling). This can be immediately seen since the peptide was absent from the treated sample, by comparing the direct and inverted experiments (label swapping). One peptide was not labeled (No labeling) and most peptides receive a partial labeling (see Figure 2b).

Figure S2 The estimated fold change is compared between the experimental spectra (red) and the simulated spectra (blue). The simulated spectra were generated as a symmetrical distribution with a standard deviation twice as large as the ones observed to allow the investigation of high fold change peptides. The experimentally observed spectra pair with a completely absent treated peptide was excluded since its theoretical fold change was –infinite.

Figure S3 When the labeling efficiency decreases the fold change error was found to increase. For a low noise level (noise ∈[0.01 0.10]) we found an almost constant or slowly increasing fold change error upon decreasing labeling efficiency, while for increased noise level the fold change error was found to increase steadily with poorer labeling efficiency.

Figure S4 The labeling efficiency estimate is generally quite reliable, but depends on the level of noise in the spectra. An interesting aspect is that on average it is overestimated for high labeling efficiencies and underestimated when it is low. The black line illustrates the ideal labeling efficiency.

Figure S5 Examples of experimentally observed spectra pairs are shown in relationship to the contour plot delineating the average fold change error given the Error/Signal-ration and labeling efficiency estimated by the method presented.
